# The Nordström Question

**DOI:** 10.3390/life13071442

**Published:** 2023-06-26

**Authors:** William D. Donachie

**Affiliations:** Institute of Cell and Molecular Biology, University of Edinburgh, Edinburgh EH9 3FD, Scotland, UK; uilleam4@gmail.com

**Keywords:** Initiation volume (*Vi*), unit length, partition

## Abstract

It is suggested that the absolute dimensions of cells of *Escherichia coli* may be set by the separation distance between newly completed sister nucleoids.

## 1. Introduction

A bit of background.

In 1967 and 1968, the “*C*+*D*” model of Steven Cooper and Charles Helmstetter provided a framework via with which to interpret measurements on the patterns of chromosomal DNA synthesis in cultures of *Escherichia coli* B/r cells, at different growth rates (at 37 °C) [1,2,3].

A possible explanation for the constancy of “C” at moderate to high growth rates was fairly clear: the rate of DNA synthesis at each replication fork could be independent of substrate concentration (i.e., “substrate saturation”) and therefore of growth rate. At that time, no explanation could be given for the growth rate independence of “*D*”, the number of minutes between the completion of the “*C*” period and the completion of cell division. Although so much is now known about the mechanism of division, the constancy of “*D*” remains a bit of a mystery (although I would guess that it has something to do with the mechanism of growth of the septal ring, and with the polymerisation of FtsZ in particular). However, what was not obvious was that which cells had in common when new rounds of chromosome replication were initiated, thus apparently initiating the cell duplication cycle. Therefore, the question to be asked was, “What do all of these cells have in common at the time of initiation of rounds of DNA replication?”.

The answer was in the library. I found the clue in a paper by Schaechter, Maaløe and Kjeldgaard [4], in which they showed that the mean dry weight/cell increased exponentially with the growth rate in asynchronous cultures of *Salmonella typhimurium* growing in “balanced” exponential growth at 37ºC on a variety of different carbon sources.

The next step was easy (having ascertained from Bacteriologist colleagues that *Salmonella typhimurium* was really much the same as *E. coli*!). Assuming that cells grow exponentially in mass over each cell cycle, and using Powell’s age distribution equation for ideal asynchronous logarithmic-phase populations [5] with Elio’s graph [4], the dry weight/cell could be calculated for cells of all growth rates at the time of the initiation of chromosome replication in each cell cycle. As it turned out, the dry weight/cell was in fact *not* the same at the time of initiation in cells at every different growth rate; however, what *was* the same for cells with doubling times between 60 min and 20 min (the shortest known doubling time for *E. coli* B/r at 37 °C) was the ratio of the cell mass to the number of chromosome origins (*oriC*) at the time every new round of chromosome replication was initiated (r.o.r.). Eureka!…sort of.

Therefore, I called this ratio (mass/*oriC*) at the start of each r.o.r., “Initiation Mass (*Mi*)” and submitted a short paper to *Nature* [6]. This paper is no more than an exercise in logic. Noone would have been less surprised than me if the measured values of cell mass at initiation proved to vary from this model, because, if nothing else, the experimental measurements of the mean cell dry weight in cultures growing at different rates were scattered around the best-fit regression line. The measured values of the *C* and *D* times were also variable. The composition of cells also changes with the growth rate. In particular, the proportion of cell mass due to ribosomes also changes dramatically with the growth rate, as Maaløe and Kjeldgaard [7] have shown; how could a cell measure its mass anyway? Models for the initiation of chromosome replication, being based on chemical interactions, of course implied that the concentration of reactants, rather than the absolute amounts, was what was important. For this reason (and assuming that cell mass and cell volume were proportional to one another), I later preferred to refer to “Initiation Volume (*Vi*)” rather than Mi.

## 2. Kurt Nordström’s Killer Question

In 2000, I retired and became interested in other things; however, annoyingly, even after 32 years, there was still no satisfactory explanation as to *how* the initiation of chromosomal DNA replication is linked to cell size. For one last try, and because there were lots of interesting new experiments, Garry Blakely and I devised yet another model for periodic initiation [8]. As everyone seemed to agree, it was clear that a critical event was the binding of the DnaA protein to *oriC;* however, although reducing the rate of production of DnaA protein does indeed delay initiation, alas increasing the production of DnaA has only a marginal effect on initiation timing. Therefore, something else must also be limiting for initiation. Our model [8] proposed that competition between DnaA-ATP protein (active form) and DnaA-ADP (inactive) for binding to *oriC*, taken together with the post-initiation inactivation of all DnaA-ATP and a transient block to reinitiation at *oriC,* could provide part of the explanation for the periodic initiation of chromosomal DNA replication linked to cell growth and size. Our model predicted a peak in the DnaA-ATP/DnaA-ADP ratio at every doubling in cell volume.

In 2002, I was invited to give that year’s Nordström Lecture in Uppsala. I presented our model and thought that the lecture had been well enough received. However, in his office afterward, Kurt Nordström asked me a question, something like this: “Tell me, Willie, what do you think determines the *actual* volume of Vi?”

I was so glad that Kurt had not asked that question during the seminar, because it left me completely flummoxed. The model (like similar previous models) did not specify the actual value of *Vi*; indeed, it would work for any arbitrary cell volume and give peaks in DnaA-ATP/DnaA-ADP at each doubling of whatever volume that was.

In June 2022, I gave a short “Zoom” presentation at a symposium in Copenhagen (“Major Ideas in Quantitative Microbial Physiology: Past, Present and Future”, organised by Suckjoon Jun) because I thought that I had finally come up with a (partial) answer to Kurt Nordström’s question. You will decide whether I have!

## 3. “Unit Length” (*L* microns) Extension between Completion of r.o.r. Defines *Vi* (Cubic Microns)

(The following idealised description of the geometry of *E. coli* cells applies only under conditions in which C + D = 40 + 20 min.)

One of the many attractions of an *E. coli* cell is its simple geometry and consistent mode of growth. To a first approximation, the cell is a cylinder with hemispherical poles. When growing under constant conditions, the cell elongates without change in the width of the cylinder. Again to a first approximation, the rate of cell elongation appears to be exponential.

This is the mode of cell growth for most if not all steady growth rates in different media. However, both the average length and average width of the growing cylinders change with the growth rate, such that the average (length/width) is constant at both low and high growth rates, as shown by Zaritsky [9]. Thus, cells growing exponentially with a growth rate of three doublings/h are, on average, twice as long and twice as wide as cells growing at one doubling/h, and the fast growing cell therefore has a volume at initiation that is four times that of the slow-growing cell).

**Figure 1:**
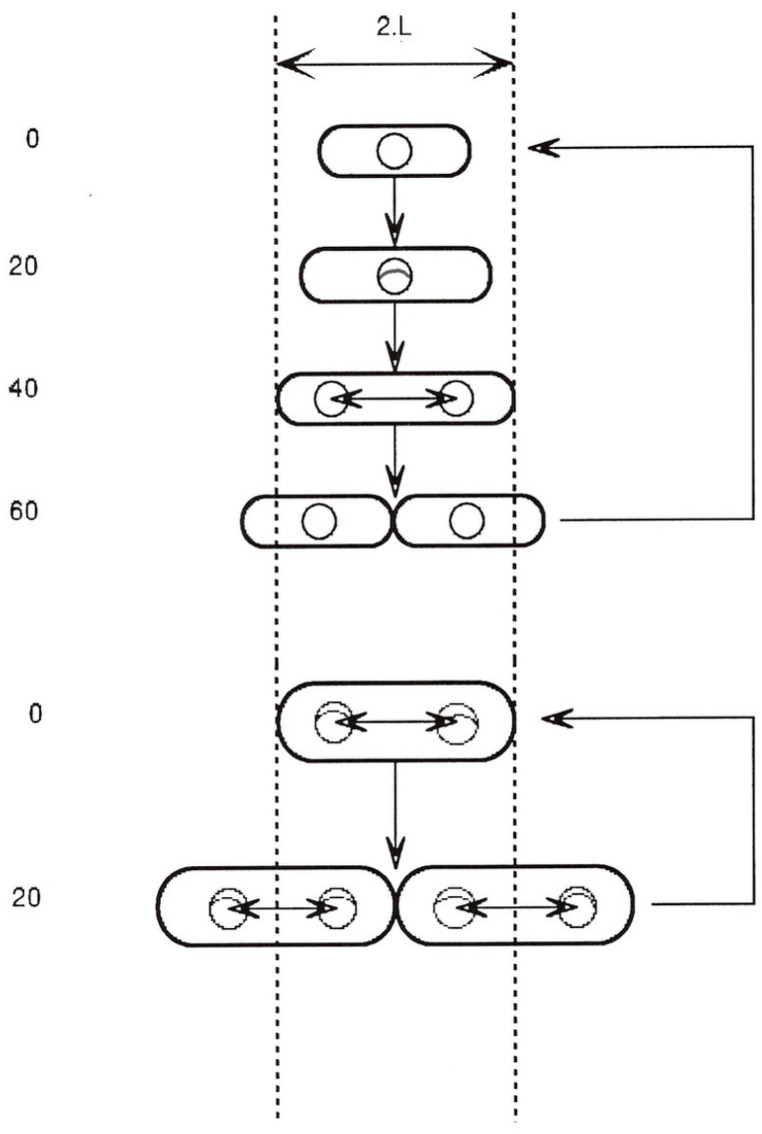
Cell size and shape, chromosome replication, and position during normal cell cycles of E. coli are discussed. The top section shows the cycle for a cell growing in balanced growth with a doubling time of 60 min, and the lower section shows the cycle for a doubling time of 20 min. Cells are drawn to scale (where 2.L, or two unit lengths, is about 2.8 microns). In balanced growth at any constant growth rate, a cell grows exponentially only by elongation, without change in width. However, average cell volume increases exponentially with growth rate [4] although the relative proportions of length to width remain constant [9]. The chromosome is drawn as a small circle (with replication forks when present), but this is not to the same scale. However, the circle is drawn with a diameter roughly equal to that of the nucleoid [10]. The time taken for each replication fork to travel from *oriC* (top of circle) to the terminus (bottom of circle) is 40 min (independent of growth rate) [1,2,3]. At all growth rates, completion of each round of chromosome replication takes place as the cell reaches a fixed length (2.L) [11,12] and sister nucleoids then separate by a fixed distance, 1.L, which positions them in the centres of the incipient sister cells [10]. Cell division also commences in the cell centre at this cell length (2.L) and takes 20 min to complete, independent of growth rate [1,2,3].

Figure 1 shows the relative proportions (length and width) of such an ideal cell at 20 min intervals while growing exponentially at either one doubling/h (top) or three doublings/h (bottom).

Despite the differences in the *average* length, width and volume, it is a curious fact that there is a point in the growth cycle when all cells have the same length (“2L”), independent of the growth rate or cell volume [11,12]. We may therefore ask whether there is anything else that takes place only at this stage in the cell growth cycle.

The figure shows three events that occur only at around this cell length:

1. Completion of a round of chromosome replication (r.o.r.) [1,2,3].
2. Relocation of sister chromosomes from the cell centre to the cell quarters [10,13].
3. Initiation of the septal ring at the mid-point between sister chromosomes [14].

If cells obey these growth rules, then it follows from geometry that *Vi* (cell volume/origin) must also be constant at the time of the initiation of each round of chromosome replication (i.e., *C* minutes earlier), independent of growth rate. Therefore, when we ask a question about some aspect of the cell’s geometry, we are also asking questions about all the others: volume is defined by length and width, and vice versa. Asking what determines *Vi* is the same as asking what determines *L*. There is little doubt in my mind that *L*, or the unit length, is a real constant independent of growth rate; it is the fixed amount by which cells extend their length between the end of one r.o.r. and the end of the next. Considered this way, we could answer the Nordström question by stating that the constancy and value (microns) of *L* implies the constancy and value (cubic microns) of *Vi*.

## 4. Does Partition Distance Govern Unit Length?

Our laboratory, like many others, searched for cell cycle genes for over 20 years. From scores of mutants, the genes and proteins specific for cell division have been successfully uncovered and yet no one (to my doubtless outdated knowledge) has found proteins in *E. coli* that are specific for chromosome partition. Mutations in a few proteins (e.g., MukB, DNA gyrase, FtsK) may interfere with sister chromosome separation but none of these proteins provide an actual partition mechanism. Yet, sister nucleoids clearly do (and logically must) move apart after replication. Moreover, they move apart, seemingly quite suddenly, by about half a cell length [10,13] (as they must for an efficient growth and division cycle); therefore, what is the hidden mechanism of partition? Alas, I do not know the answer. However, I remember discussing this problem with Vic Norris in Paris (in a gym!) sometime in the late 1980s. Vic’s suggestion was along the lines that, “If sister DNA molecules each have a net negative charge, then they ought to repel one another”. In a narrow cylinder such as an *E. coli* cell, the direction of movement would necessarily be in the long axis, while the inverse square law plus the viscosity of the cytoplasm would determine the average distance moved. Could this distance correspond to “*L*”? If it did, then Kurt Nordström’s question would be answered: “The invariant increase in cell length/cycle (*L* microns), and hence the value of *Vi* (cubic microns/*oriC*) would have evolved by Natural Selection to fit the partition distance (*L* microns) which in turn is determined by Physics.”

It has not escaped my notice that the above suggestion raises any number of questions! e.g.,

1. How is a single replicating molecule of DNA kept together as a single nucleoid (by MukB and Gyrase), but suddenly changed to two mutually repellent nucleoids at the completion of replication? (If it is).
2. How is the extent of cell elongation between the completion of each r.o.r. fixed independent of the rate of volume growth?
3. Am I describing the “adder” phenomenon in another way?
4. Gram-positive rods, such as *Bacillis subtilis*, which do not change width with the growth rate, would require a different process to ensure that sister chromosomes are located in sister cell centres. These organisms, unlike *E. coli*, possess *par* genes.
5. And so on.

## 5. Thanks and Apologies

When I retired, I deliberately stopped reading about the cell cycle, because by that time, it looked as if most cell-division-specific genes and proteins had been identified and that the main outline of the *E. coli* cell cycle was clear enough. Job done! I blush as I write that but it is what I did. Therefore, all the marvellous new technologies and amazing new insights passed me by, like Rip van Winkle. Nearly 20 years, later I received a phone call from Johan Elf, who kindly thought that I might like to know that *Vi* really is constant. That woke me up briefly, but I went back to sleep almost at once (albeit with a smile on my face!); this was until Suckjoon Jun and his colleagues sent me a draft of their great review [15] and the extent of the whole rejuvenated world of *E. coli* cell cycle research was revealed to me! And then I had to think of something new to say in my little talk for the Copenhagen meeting, and I remembered what Kurt Nordström had asked me 20 years before.

I hope I have not made any embarrassing errors in fact or logic, or tediously reinvented the wheel, but I have enjoyed trying to answer Kurt’s question. The regret of course is that I cannot discuss it with him (although I am sure that he would have had some other killer question!). However, I am glad that I can thank Johan Elf, Ariel Amir (who visited me here, after I missed his seminar!), Arieh Zaritsky (who, amongst other kindnesses, asked me for this contribution) and Suckjoon Jun, for really waking me up!

My final thanks go to Vic Norris, not only for providing the electrifying idea about partition, but also for offering to get this little essay into some sort of publishable form.
